# Vectorial Image Representation on the Texture Space (VIR-TS) Applied to RGB Image Classification: Face Recognition

**DOI:** 10.3390/jimaging12070295

**Published:** 2026-07-02

**Authors:** Héctor Guillen-Bonilla, José Trinidad Guillen-Bonilla, Maricela Jiménez-Rodríguez, Alex Guillen-Bonilla, Jorge Aguilar-Santiago, Lucía Ivonne Juárez-Amador, Antonio Casillas Zamora

**Affiliations:** 1 Departamento de Ingeniería de Proyectos, Centro Universitario de Ciencias Exactas e Ingenierías, Universidad de Guadalajara, Blvd. M. García Barragán 1421, Guadalajara 44410, Jalisco, Mexico; hector.guillen1775@academicos.udg.mx (H.G.-B.); lucia.juarez@academicos.udg.mx (L.I.J.-A.); 2 Departamento de Electro-Fotónica, Centro Universitario de Ciencias Exactas e Ingenierías, Universidad de Guadalajara, Blvd. M. García Barragán 1421, Guadalajara 44410, Jalisco, Mexico; antonio.czamora@academicos.udg.mx; 3 Departamento de Ciencias Básicas, Centro Universitario de la Ciénega, Universidad de Guadalajara, Av. Universidad No. 1115, Lindavista, Ocotlán 47810, Jalisco, Mexico; maricela.jrodriguez@academicos.udg.mx; 4 Departamento de Ciencias Computacionales e Ingenierías, Centro Universitario de los Valles, Universidad de Guadalajara, Carretera Guadalajara-Ameca Km. 45.5, Ameca 46600, Jalisco, Mexico; alex.guillen@academicos.udg.mx; 5 Departamento de Ciencias Tecnológicas, Centro Universitario de la Ciénega, Universidad de Guadalajara, Av. Universidad No. 1115, Lindavista, Ocotlán 47810, Jalisco, Mexico; jorge.asantiago@academicos.udg.mx

**Keywords:** RGB image, Vectorial Image Representation on the Texture Space (VIR-TS) transform, multi-class classifier, texture space, classification efficiency

## Abstract

In this paper, an RGB image with S is separated by its channels, obtaining an image in each color channel $S_{R}$, $S_{G}$ and $S_{B}$. The Vectorial Image Representation on the Texture Space (VIR-TS) transform is calculated for each channel; ergo, each image is represented with a given vector, $S_{R}\to \;{\vec{C}}_{R}$, $S_{G}\to {\vec{C}}_{G}$ and $S_{B}\to {\vec{C}}_{B}$. Employing the ${\vec{C}}_{R}$, ${\vec{C}}_{G}$, and ${\vec{C}}_{B}$ vectors in a multi-class classifier, a database of RGB images was identified with the aim of verifying the classification efficiency of the VIR-TS transform. Based on the experimental results, the VIR-TS technique presents high efficiency when the noise is not added to the class and when the signal-to-noise ratio is high. For both instances, the efficiency presented is 100%. Nonetheless, if the class noise is high, the efficiency diminishes from 100%, to 95%, to 90% until it decreases to 10%. Based on the results obtained, the VIR-TS transform can be efficiently applied for the development of security systems and access control and can also be implemented in computer vision systems for medical diagnosis, drones, etc.

## 1. Introduction

Computer vision is frequently employed to identify points of interest within images, applied in facial recognition, medical areas or in the classification of any type of object. To perform these tasks, it is necessary to determine the texture characteristics of images with regions of interest. This task is commonly performed with texture descriptors that work closely with statistical classifiers or neural networks, and these are in charge of identifying the class to which the region of interest being studied belongs. Texture descriptors and statistical classifiers or neural networks have been deployed in previous papers to cease identity theft by applying facial recognition [1,2,3]. The biometric identification of people is performed by facial recognition through a multilevel hierarchy representation that applies a set of local classifiers to make decisions [4]. One of the most commonly used descriptors is the Local Binary Pattern (LBP) [5,6,7], which works with binary patterns; it is applied by dividing the data in three arrays, where two remain fixed, to carry out an in-depth analysis with the intention of optimizing them considering the decomposition of singular values [8]. A study was performed to compare the characteristics of 10 texture descriptors employed for facial and palmprint recognition [9]. A new method to detect facial expressions was presented where Local Binary Patterns (LBP), Local Ternary Patterns (LTP) and Completed Local Binary Patterns (CLBP) were deployed with a Convolutional Neural Network [10]. An optimized Histogram of Oriented Gradients (HOG) algorithm is applied to extract facial characteristics along with a Tree-structured Parzen Estimator (TPE)-based Bayesian optimization technique [11]. An MB-ZZLBP descriptor was deployed for face analysis to perform facial recognition with SVM and NN [12]. Multi-Resolution Multi-Threshold Local Binary Patterns (MRMT-LBPs) were applied to emphasize shades of gray, and the authors of that study proposed systematically generating multiple LBP representations to perform facial recognition [13]. Even employing excellent descriptors and texture classifiers, there exist great challenges that can affect the performance of facial recognition methods, such as lighting variance in the photographs, pose and facial expressions, occlusion or data loss; additionally, there could be disturbances or failures in facial recognition techniques when the images present noise. To mitigate these problems, modifications to the LBP method were proposed to improve the virtual electrical binary pattern descriptor against noise [14], and Support Vector Machine (SVM) was applied for classification. Furthermore, a multi-level adaptative mining model was applied for filtering and a pyramidal block was used to capture the inconsistent characteristics provoked by regional noise [15]. To perform facial expression recognition, a hybrid framework was proposed applying MobileNetV2 deep learning and LBP and HOG descriptors [16]. Facial recognition is employed to determine the drowsiness state of drivers using the HOG descriptor and SVM classifier [17]. Variations in the LBP texture descriptor were performed to generate a Multiscale Discriminative Binary Pattern (MDBP), which was deployed for facial recognition; the central pixel is replaced by a threshold value generated with the mean and the median [18]. A paper presented an investigation of how noise can propagate from the source domain to the target domains where it cannot be detected, causing a decrement in performance [19]; noise alters image quality, which in turn influences facial recognition [20,21]. Without a doubt, any degradation present in facial recognition techniques when images present noise is a subject of great interest.

In reference [22], the Vectorial Image Representation on the Texture Space (VIR-TS) transform was described and applied. The VIR-TS transform extracts the local texture characteristics of a grayscale image, and these are represented in the texture space as vectors on which the direction and magnitude depend on the image under study. The vector was employed in a multi-class classifier where each class belonged to a tree trunk bearing a very high classification efficiency, reaching up to 100% when images had no additional noise. In this paper, facial recognition in RGB images is performed considering their local texture characteristics. The RGB images are analyzed for each color channel and the Vectorial Image Representation on the Texture Space (VIR-TS) transform is applied for extraction of the local texture characteristics. Each class is represented on the texture space by three random vectors, ${\vec{C}}_{R}$, ${\vec{C}}_{G}$, and ${\vec{C}}_{B}$, where each vector corresponds to a channel and its magnitude and phase are functions of the texture. These vectors are deployed in a multi-class classifier to measure the similarities with the classes in the texture space through the projection of a prototype vector and a test vector. Using a facial image database, the images are identified with and without added noise to their respective classes. The VIR-TS transform yields an efficiency of 100% if and only if the classes bear no added noise and if the signal-to-noise ratio is very high in the class. Otherwise, if the signal-to-noise ratio is small, the VIR-TS transform becomes less efficient at identifying the class.

Taking in consideration these results, the VIR-TS transform can be employed to build highly efficient machine vision systems. These systems would be based on the identification of textural characteristics and they could be applied in several fields, for example, medical diagnosis, security, access control, analysis of medical images, and the location of missing persons, among other applications.

## 2. Theory

### 2.1. Previous Work

The Vectorial Image Representation on the Texture Space (VIR-TS) transform represents an **S** grayscale digital image through a $\vec{C}$ vector in the texture space [22]. In the VIR-TS transformation, the grayscale image in a random $S=\{s_{mn}\}\;(m=1,2,\ldots ,M;n=1,2,\ldots ,N)$ array, with a size of M×N pixels and where every position has a discrete pattern $P=\{p_{i,j}\}\;(I=1,2,3;J=1,2,3)$, is detected by an observation window with a size of W=I×J=3×3. Each P pattern is employed to calculate a texture unit vector $\vec{T}$, and thus all texture unit vectors are measured to estimate the $\vec{C}$ vector.

#### 2.1.1. Texture Unit

Given a grayscale image $S=\{s_{mn}\}\;(m=1,2,\ldots ,M;n=1,2,\ldots ,N)$ where a random pattern is detected with an I×J=3×3 mobile observation window $P=\{p_{i,j}\}\;(I=1,2,3;J=1,2,3)$, the texture unit is calculated in the VIR-TS transform, and next a homogeneous equation system is considered [22]:

(1) $$C_{P}T=0\;\;\;⟹\;\;\;\;\left[\begin{array}{ccc} p_{11} & p_{12} & p_{13}\\ p_{21} & p_{22} & p_{23}\\ \;p_{31} & p_{32} & p_{33} \end{array}\right]\left(\begin{array}{c} t_{1}\\ t_{2}\\ t_{3} \end{array}\right)=\left[\begin{array}{c} 0\\ 0\\ 0 \end{array}\right]\;\;\;⟹\;\begin{array}{c} p_{11}t_{1}+p_{12}t_{2}+p_{13}t_{3}=0\\ p_{21}t_{1}+p_{22}t_{2}+p_{23}t_{3}=0\\ p_{31}t_{1}+p_{32}t_{2}+p_{33}t_{3}=0 \end{array}$$

where $C_{P}=\left[\begin{array}{ccc} p_{11} & p_{12} & p_{13}\\ p_{21} & p_{22} & p_{23}\\ \;p_{31} & p_{32} & p_{33} \end{array}\right]$ is called the coefficient matrix of the homogeneous linear system, represented with a 3×3 matrix of real elements, and $T=\left[\begin{array}{c} t_{1}\\ t_{2}\\ t_{3} \end{array}\right]$ is called a texture unit vector. The homogeneous equation system yields a trivial solution $t_{1}=0$, $t_{2}=0$, $t_{3}=0$, though this solution is not functional for texture recognition. Consequently, a non-trivial solution must be obtained. Based on fundamental linear algebra concepts, a non-trivial solution can be found for an equation system if and only if the matrix determinant is equal to zero, $det\left|C_{P}\right|=0$; ergo

(2) $$det\left|C_{P}\right|=det\left|\begin{array}{ccc} p_{11} & p_{12} & p_{13}\\ p_{21} & p_{22} & p_{23}\\ \;p_{31} & p_{32} & {Kp}_{33} \end{array}\right|=0$$

where the K term must satisfy condition (2). Thus, our problem is to find the value of K as a function of the elements of the pattern P. To find the value of K, the determinant of the matrix $C_{P}$ is calculated and the result is set equal to zero,

(3) $$det\left|\begin{array}{ccc} p_{11} & p_{12} & p_{13}\\ p_{21} & p_{22} & p_{23}\\ \;p_{31} & p_{32} & {Kp}_{33} \end{array}\right|={Kp}_{33}p_{22}p_{11}+p_{12}p_{23}p_{31}+p_{13}p_{21}p_{32}-p_{13}p_{22}p_{31}-{Kp}_{33}p_{12}p_{21}-p_{11}p_{23}p_{32}=0$$

From this result the parameter K can be determined, obtaining

(4) $$K=\frac{p_{13}p_{22}p_{31}+p_{11}p_{23}p_{32}-p_{12}p_{23}p_{31}-p_{13}p_{21}p_{32}}{p_{33}p_{22}p_{11}-p_{33}p_{12}p_{21}}$$

Calculating the parameter K, it is introduced to the equation system:

(5) $$\begin{array}{c} p_{11}t_{1}+p_{12}t_{2}+p_{13}t_{3}=0\\ p_{21}t_{1}+p_{22}t_{2}+p_{23}t_{3}=0\\ p_{31}t_{1}+p_{32}t_{2}+Kp_{33}t_{3}=0 \end{array}$$

The solution of the homogeneous equation system can be calculated via parameterization, obtaining [22]

(6) $$\infty -soluciones\left\{\begin{array}{cc} \begin{array}{c} t_{1}=\frac{p_{13}p_{22}-p_{23}p_{12}}{p_{11}p_{22}-p_{12}p_{21}}\lambda \\ t_{2}=\frac{p_{1,1}p_{2,3}-{p_{21}p}_{13}}{p_{11}p_{22}-p_{12}p_{21}}\lambda \\ t_{3}=\lambda \;\;\;\;\;\;\;\;\;\;\;\;\;\;\; \end{array} & \lambda \in R \end{array}\right..$$

Observing expression (6), for each real value of λ, a unique solution from the infinite solutions is found. Based on Equations (1) and (6), the texture unite vector can be defined by

(7) $$T=\left[\begin{array}{c} t_{1}\\ t_{2}\\ t_{3} \end{array}\right]=\left[\begin{array}{c} \frac{p_{13}p_{22}-p_{23}p_{12}}{p_{11}p_{22}-p_{12}p_{21}}\lambda \\ \frac{p_{11}p_{23}-{p_{21}p}_{13}}{p_{11}p_{22}-p_{12}p_{21}}\lambda \\ \lambda \end{array}\right]$$

And its cartesian representation yields the form

(8) $$\overset{⃑}{T}=t_{1}\;{\hat{u}}_{1}+t_{2}\;{\hat{u}}_{2}+t_{3}\;{\hat{u}}_{3}=\frac{p_{13}p_{22}-p_{23}p_{12}}{p_{11}p_{22}-p_{12}p_{21}}\lambda {\hat{u}}_{1}+\frac{p_{11}p_{23}-{p_{21}p}_{13}}{p_{11}p_{22}-p_{12}p_{21}}\lambda \;{\hat{u}}_{2}+\lambda {\hat{u}}_{3}.$$

where ${\hat{u}}_{1}$, ${\hat{u}}_{2}$,$\;{\hat{u}}_{3}$ are the unitary vectors of a three-dimensional rectangular coordinate system. Parting from (8), the scalars $\frac{p_{13}p_{22}-p_{23}p_{12}}{p_{11}p_{22}-p_{12}p_{21}}\lambda ,\frac{p_{11}p_{23}-{p_{21}p}_{13}}{p_{11}p_{22}-p_{12}p_{21}}\lambda$ and λ are the components of vector $\overset{⃑}{T}$ with $u_{1},u_{2},u_{3}$ as their directions. Lastly, in Equation (8), the magnitude of vector $\overset{⃑}{T}$ is calculated by

(9) $$\left|\overset{⃑}{T}\right|=\sqrt{{\left(\frac{p_{13}p_{22}-p_{23}p_{12}}{p_{11}p_{22}-p_{12}p_{21}}\lambda \right)}^{2}+{\left(\frac{p_{11}p_{23}-{p_{21}p}_{13}}{p_{11}p_{22}-p_{12}p_{21}}\lambda \right)}^{2}+\lambda^{2}}.$$

and their cosines are

(10) $$\begin{array}{c} cos\alpha =\frac{\frac{p_{13}p_{22}-p_{23}p_{12}}{p_{11}p_{22}-p_{12}p_{21}}\lambda }{\left|\overset{⃑}{T}\right|}\\ cos\beta =\frac{\frac{p_{11}p_{23}-{p_{21}p}_{13}}{p_{11}p_{22}-p_{12}p_{21}}\lambda }{\left|\overset{⃑}{T}\right|}\\ cos\gamma =\frac{\lambda }{\left|\overset{⃑}{T}\right|} \end{array}.$$

The following condition is satisfied:

(11) $${cos}^{2}\alpha +{cos}^{2}\beta +{cos}^{2}\gamma =1,$$

where $\left|\overset{⃑}{T}\right|$ is the magnitude of vector $\overset{⃑}{T}$. In simpler terms, the texture unit $\overset{⃑}{T}$ is a radius vector that goes from the origin to the coordinates $t_{1}=\frac{p_{13}p_{22}-p_{23}p_{12}}{p_{11}p_{22}-p_{12}p_{21}}\lambda ,\;t_{2}=\frac{p_{11}p_{23}-{p_{21}p}_{13}}{p_{11}p_{22}-p_{12}p_{21}}\lambda ,t_{3}=\lambda$.

#### 2.1.2. Representation of a Gray-Level Image in the Texture Space

Given an image S with a size of M×N pixels and if this image is studied applying a I×J window size, then there are $\left(M-I+1\right)\times \left(N-J+1\right)$ patterns P. Conversely, knowing that a texture unit vector $\overset{⃑}{T}$ is calculated for each pattern based in a homogenous system equation, it follows that if the S image is analyzed with the observation window, for the n−th pattern $P_{n}$, where $\left(n=1,2,\ldots ,N_{P}=\left(M-I+1\right)\times \left(N-J+1\right)\right)$, the n−th texture unit ${\vec{T}}_{n}$ (radius vector) is calculated. Consequently, the S image can be expressed through a series of radius vectors, such that by summing their components on the texture space, the S gray-level image is represented by a $\vec{C}$ vector defined as

(12) $$\vec{C}=a_{1}{\hat{u}}_{1}+a_{2}{\hat{u}}_{2}+a_{3}{\hat{u}}_{3},$$

where ${\hat{u}}_{1},{\hat{u}}_{2},{\hat{u}}_{3}$ are the unitary vectors and $a_{1},a_{2},a_{3}$ are the components, and they are calculated by

(13) $$\begin{array}{c} a_{1}=\sum_{n=1}^{N_{P}}t_{1n}\\ a_{2}=\sum_{n=1}^{N_{P}}t_{2n}\\ a_{3}=\sum_{n=1}^{N_{P}}t_{3n} \end{array},$$

**Figure 1:**
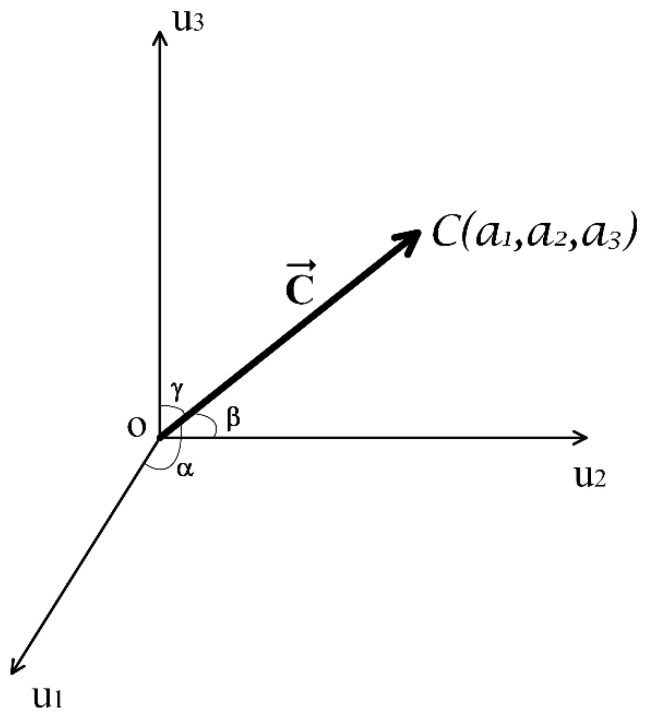
Graphic representation of the texture vector $\vec{C}$ with its cosines.

$N_{P}$ is the total patterns found in the digital image. $A=\left(M-I+1\right)\times \left(N-J+1\right)$ and $t_{1n}$, $t_{2n}$,$\;t_{3}$ are the n−th component of the elements for $t_{1}$,$\;t_{2}$,$\;t_{3}$, respectively. Figure 1 illustrates the $\vec{C}$ vector.

Based in Figure 1, the $\vec{C}$ vector has a magnitude of

(14) $$\left|\vec{C}\right|=\sqrt{a_{1}^{2}+a_{2}^{2}+a_{3}^{2}},$$

The cosines are

(15) $$\begin{array}{c} cos\alpha =\frac{a_{1}}{\left|\vec{C}\right|}\\ cos\beta =\frac{a_{2}}{\left|\vec{C}\right|}\\ cos\gamma =\frac{a_{3}}{\left|\vec{C}\right|} \end{array}$$

And it is fulfilled by

(16) $${cos}^{2}\left(\frac{a_{1}}{\left|\vec{C}\right|}\right)+{cos}^{2}\left(\frac{a_{2}}{\left|\vec{C}\right|}\right)+{cos}^{2}\left(\frac{a_{3}}{\left|\vec{C}\right|}\right)=1.$$

Considering the analysis, the grayscale image S can be represented like a radius vector $\vec{C}$ in the texture space, the direction and magnitude of which depend on the randomness of the digital image.

### 2.2. VIR-TS Applied to RGB Images

As it is known, digital images can be in color, grayscale or binary. All three types are commonly employed in texture recognition. In particular, RGB images are conformed by three channels, where each channel corresponds to a basic color: red, green and blue. When these images are classified based on their local texture, their identification is implemented by performing the recognition for each color channel. The recognition efficiency depends on the texture extraction technique. Taking this into consideration, in this section, the recognition of RGB images is implemented with the VIR-TS technique. To achieve this, the procedure described in Section 2.1 is applied to each color channel, conforming the RGB image.

#### 2.2.1. Texture Unit Definition for Each Color Channel

In this section, our proposal is that the color image S can be separated by channel; thus, $S_{R}$ is the red channel image, $S_{G}$ corresponds to the green channel image and $S_{B}$ contains the blue channel image. The three images $S_{R}$, $S_{G}$ and $S_{B}$ have a size of M×N $\left(m=1,2,3,\ldots ,M;n=1,2,3,\ldots ,N\right)$ pixels, and each pixel yields a value within the interval of 0 to 255. Under these conditions, the Vectorial Image Representation on the Texture Space (VIR-TS) transform can be applied to each channel of the RGB image. Developing a process for each color channel of an RGB is performed following the approach in Section 2.1, Therefore, if the $W_{R}$, $W_{G}$, $W_{B}$ windows detect $R=\left[\begin{array}{ccc} r_{11} & r_{12} & r_{13}\\ r_{21} & r_{22} & r_{23}\\ \;r_{31} & r_{32} & r_{33} \end{array}\right]$, $G=\left[\begin{array}{ccc} g_{11} & g_{12} & g_{13}\\ g_{21} & g_{22} & g_{23}\\ \;g_{31} & g_{32} & g_{33} \end{array}\right]$, $B=\left[\begin{array}{ccc} b_{11} & b_{12} & b_{13}\\ b_{21} & b_{22} & b_{23}\\ \;b_{31} & b_{32} & b_{33} \end{array}\right]$ patterns from the RGB image, then the texture unit for each channel can be defined by a homogenous linear equation system [22]:

(17) $$RT_{R}=0⟹\left[\begin{array}{ccc} r_{11} & r_{12} & r_{13}\\ r_{21} & r_{22} & r_{23}\\ \;r_{31} & r_{32} & r_{33} \end{array}\right]\left(\begin{array}{c} t_{r1}\\ t_{r2}\\ t_{r3} \end{array}\right)=\left[\begin{array}{c} 0\\ 0\\ 0 \end{array}\right]$$

(18) $$GT_{G}=0⟹\left[\begin{array}{ccc} g_{11} & g_{12} & g_{13}\\ g_{21} & g_{22} & g_{23}\\ \;g_{31} & g_{32} & g_{33} \end{array}\right]\left(\begin{array}{c} t_{g1}\\ t_{g2}\\ t_{g3} \end{array}\right)=\left[\begin{array}{c} 0\\ 0\\ 0 \end{array}\right]$$

(19) $$BT_{B}=0⟹\left[\begin{array}{ccc} b_{11} & b_{12} & b_{13}\\ b_{21} & b_{22} & b_{23}\\ \;b_{31} & b_{32} & b_{33} \end{array}\right]\left(\begin{array}{c} t_{b1}\\ t_{b2}\\ t_{b3} \end{array}\right)=\left[\begin{array}{c} 0\\ 0\\ 0 \end{array}\right]$$

Equation (17) is the texture unit definition for the red channel: $T_{R}=\left(\begin{array}{c} t_{r1}\\ t_{r2}\\ t_{r3} \end{array}\right)$ is the unity, and the components are $t_{r1},t_{r2},t_{r3}$. Equation (18) is the texture unit definition for the green channel: $T_{G}=\left(\begin{array}{c} t_{g1}\\ t_{g2}\\ t_{g3} \end{array}\right)$ is the unity, and the components are $t_{g1},t_{g2},t_{g3}$. Finally, Equation (19) is the texture unit definition for the blue channel: $T_{B}=\left(\begin{array}{c} t_{b1}\\ t_{b2}\\ t_{b3} \end{array}\right)$ is the unity and the components are $t_{b1},t_{b2},t_{b3}$. Analyzing Equations (17)–(19), each color channel is analyzed independently; thus, the condition $T_{R}\neq T_{G}\neq T_{B}$ is satisfied because each color channels bear different intensity values in their pixels due to the random nature of the light source. From Equations (17)–(19), the texture unit definition is fundamental in a homogeneous equation system with a trivial solution defined by $t_{r1}=0,\;t_{r2}=0,t_{r3}=0$ (red channel), $t_{g1}=0,\;t_{g2}=0,t_{g3}=0$ (green channels) and $t_{b1}=0,t_{b2}=0,t_{b3}=0$ (blue channel). However, this trivial solution it not applicable in our texture recognition problem. Consequently, it is proposed to find an infinite solution for our homogenous equation system and, afterwards, find a unique solution based on the resolution of the equation system through parameterization. As a first step, the equation systems (17)–(19) are structured as follows:

(20) $$RT_{R}=0⟹\left[\begin{array}{ccc} r_{11} & r_{12} & r_{13}\\ r_{21} & r_{22} & r_{23}\\ \;r_{31} & r_{32} & K_{R}r_{33} \end{array}\right]\left(\begin{array}{c} t_{r1}\\ t_{r2}\\ t_{r3} \end{array}\right)=\left[\begin{array}{c} 0\\ 0\\ 0 \end{array}\right]$$

(21) $$GT_{G}=0⟹\left[\begin{array}{ccc} g_{11} & g_{12} & g_{13}\\ g_{21} & g_{22} & g_{23}\\ \;g_{31} & g_{32} & K_{G}g_{33} \end{array}\right]\left(\begin{array}{c} t_{g1}\\ t_{g2}\\ t_{g3} \end{array}\right)=\left[\begin{array}{c} 0\\ 0\\ 0 \end{array}\right]$$

(22) $$BT_{B}=0⟹\left[\begin{array}{ccc} b_{11} & b_{12} & b_{13}\\ b_{21} & b_{22} & b_{23}\\ \;b_{31} & b_{32} & K_{B}b_{33} \end{array}\right]\left(\begin{array}{c} t_{b1}\\ t_{b2}\\ t_{b3} \end{array}\right)=\left[\begin{array}{c} 0\\ 0\\ 0 \end{array}\right]$$

where $K_{R},\;K_{G},K_{B}$ are constant values to be determined such that the determinants of the systems of equations are equal to zero:

(23) $$\begin{array}{c} det\left\lceil R\right\rceil =det\left\lceil \begin{array}{ccc} r_{11} & r_{12} & r_{13}\\ r_{21} & r_{22} & r_{23}\\ \;r_{31} & r_{32} & K_{R}r_{33} \end{array}\right\rceil =0\\ det\left\lceil G\right\rceil =det\left\lceil \begin{array}{ccc} g_{11} & g_{12} & g_{13}\\ g_{21} & g_{22} & g_{23}\\ \;g_{31} & g_{32} & K_{G}g_{33} \end{array}\right\rceil =0\\ det\left\lceil B\right\rceil =det\left\lceil \begin{array}{ccc} b_{11} & b_{12} & b_{13}\\ b_{21} & b_{22} & b_{23}\\ \;b_{31} & b_{32} & K_{B}b_{33} \end{array}\right\rceil =0 \end{array}.$$

Resolving the determinants, we obtain

(24) $$\begin{array}{c} det\left\lceil \begin{array}{ccc} r_{11} & r_{12} & r_{13}\\ r_{21} & r_{22} & r_{23}\\ \;r_{31} & r_{32} & K_{R}r_{33} \end{array}\right\rceil =0\\ =K_{R}\left(r_{33}r_{22}r_{11}-r_{33}r_{12}r_{21}\right)+r_{12}r_{23}r_{31}+r_{13}r_{21}r_{32}-r_{13}r_{22}r_{31}-r_{11}r_{23}r_{32}=0\\ \begin{array}{c} det\left\lceil \begin{array}{ccc} g_{11} & g_{12} & g_{13}\\ g_{21} & g_{22} & g_{23}\\ \;g_{31} & g_{32} & K_{G}g_{33} \end{array}\right\rceil =0\\ =K_{G}\left(g_{33}g_{22}g_{11}-g_{33}g_{12}g_{21}\right)+g_{12}g_{23}g_{31}+g_{13}g_{21}g_{32}-g_{13}g_{22}g_{31}-g_{11}g_{23}g_{32}=0\\ \begin{array}{c} det\left\lceil \begin{array}{ccc} b_{11} & b_{12} & b_{13}\\ b_{21} & b_{22} & b_{23}\\ \;b_{31} & b_{32} & K_{B}b_{33} \end{array}\right\rceil =0\\ =K_{B}\left(b_{33}b_{22}b_{11}-b_{33}b_{12}b_{21}\right)+b_{12}b_{23}b_{31}+b_{13}b_{21}b_{32}-b_{13}b_{22}b_{31}-b_{11}b_{23}b_{32}=0 \end{array} \end{array} \end{array}$$

From Equation (24), the constant values, $K_{R},\;K_{G},K_{B}$, are calculated with

(25) $$\begin{array}{c} K_{R}=\frac{r_{13}r_{22}r_{31}+r_{11}r_{23}r_{32}-r_{12}r_{23}r_{31}-r_{13}r_{21}r_{32}}{r_{33}r_{22}r_{11}-r_{33}r_{12}r_{21}}\\ K_{G}=\frac{g_{13}g_{22}g_{31}+g_{11}g_{23}g_{32}-g_{12}g_{23}g_{31}-g_{13}g_{21}g_{32}}{g_{33}g_{22}g_{11}-g_{33}g_{12}g_{21}}\\ K_{B}=\frac{b_{13}b_{22}b_{31}+b_{11}b_{23}b_{32}-b_{12}b_{23}b_{31}-b_{13}b_{21}b_{32}}{b_{33}b_{22}b_{11}-b_{33}b_{12}b_{21}} \end{array}$$

Once the constant values are known, the system of Equations (20)–(22) can be expressed by

(26) $$\begin{array}{c} r_{11}t_{r1}+r_{12}t_{r2}+r_{13}t_{r3}=0\\ r_{21}t_{r1}+r_{22}t_{r2}+r_{23}t_{r3}=0\\ r_{31}t_{r1}+r_{32}t_{r2}+K_{R}r_{33}t_{r3}=0 \end{array}$$

(27) $$\begin{array}{c} \begin{array}{c} g_{11}t_{g1}+g_{12}t_{g2}+g_{13}t_{g3}=0\\ g_{21}t_{g1}+g_{22}t_{g2}+g_{23}t_{g3}=0\\ g_{31}t_{r1}+g_{32}t_{r2}+K_{G}g_{33}t_{r3}=0 \end{array} \end{array}$$

(28) $$\begin{array}{c} b_{11}t_{b1}+b_{12}t_{b2}+b_{13}t_{b3}=0\\ b_{21}t_{b1}+b_{22}t_{b2}+b_{23}t_{b3}=0\\ b_{31}t_{b1}+b_{32}t_{b2}+K_{B}b_{33}t_{b3}=0 \end{array}$$

If the constants indicated in (25) are taken into consideration in the homogenous system of Equations (26)–(28), consequently, there are infinite solutions for each equation system. To find a unique solution, the system of equations can be resolved through parameterization, as was done in Reference [22], in a manner such that we reach

(29) $$\infty -solutions\left\{\begin{array}{cc} \begin{array}{c} t_{r1}=\frac{r_{13}r_{22}-r_{23}r_{12}}{r_{11}r_{22}-r_{12}r_{21}}\lambda \\ t_{r2}=\frac{r_{11}r_{23}-r_{21}r_{13}}{r_{11}r_{22}-r_{12}r_{21}}\lambda \\ t_{r3}=\lambda \;\;\;\;\;\;\;\;\;\;\;\;\;\; \end{array} & \lambda \in R \end{array}\right.$$

(30) $$\infty -solutions\left\{\begin{array}{cc} \begin{array}{c} t_{g1}=\frac{g_{13}g_{22}-g_{23}g_{12}}{g_{11}g_{22}-g_{12}g_{21}}\lambda \\ t_{g2}=\frac{g_{11}g_{23}-g_{21}g_{13}}{g_{11}g_{22}-g_{12}g_{21}}\lambda \\ t_{g3}=\lambda \;\;\;\;\;\;\;\;\;\;\;\;\;\; \end{array} & \lambda \in R \end{array}\right.$$

(31) $$\infty -solutions\left\{\begin{array}{cc} \begin{array}{c} t_{b1}=\frac{b_{13}b_{22}-b_{23}b_{12}}{b_{11}b_{22}-b_{12}b_{21}}\lambda \\ t_{b2}=\frac{b_{11}b_{23}-b_{21}b_{13}}{b_{11}b_{22}-b_{12}b_{21}}\lambda \\ t_{b3}=\lambda \;\;\;\;\;\;\;\;\;\;\;\;\;\; \end{array} & \lambda \in R \end{array}\right.$$

From (29)–(31), for each λ value, there is a unique solution for the homogeneous equation system. In this way, in the texture space, each texture unit calculated for each color channel is represented like a radio vector conformed by three components and then the texture unit can be defined in vector form as follows:

(32) $${\overset{⃑}{T}}_{R}=t_{r1}{\hat{u}}_{1}+t_{r2}{\hat{u}}_{2}+{\lambda \hat{u}}_{3}$$

(33) $${\overset{⃑}{T}}_{G}=t_{g1}\;{\hat{u}}_{1}+t_{g2}{\hat{u}}_{2}+{\lambda \hat{u}}_{3}.$$

(34) $${\overset{⃑}{T}}_{B}=t_{b1}\;{\hat{u}}_{1}+t_{b2}\;{\hat{u}}_{2}+\lambda \;{\hat{u}}_{3}$$

Considering this, when an RGB image is analyzed with the VIR-TS transform, the texture unit is constituted by three vectors, ${\overset{⃑}{T}}_{R}$, ${\overset{⃑}{T}}_{G}$, ${\overset{⃑}{T}}_{B}$, where their magnitude and direction depend on all elements in the patterns.

#### 2.2.2. Magnitude and Cosines

Once the texture unit has been defined in vector form for each channel (see Equations (32)–(34)), its magnitude and phase can be measured through

(35) $$\left|{\overset{⃑}{T}}_{R}\right|=\sqrt{{\left(t_{r1}\right)}^{2}+{\left(t_{r2}\right)}^{2}+\lambda^{2}}$$

(36) $$\left|{\overset{⃑}{T}}_{G}\right|=\sqrt{{\left(t_{g1}\right)}^{2}+{\left(t_{g2}\right)}^{2}+\lambda^{2}}.$$

(37) $$\left|{\overset{⃑}{T}}_{B}\right|=\sqrt{{\left(t_{b1}\right)}^{2}+{\left(t_{b2}\right)}^{2}+\lambda^{2}}$$

where $\left|{\overset{⃑}{T}}_{R}\right|$, $\left|{\overset{⃑}{T}}_{G}\right|$, $\left|{\overset{⃑}{T}}_{B}\right|$ are the magnitude for each texture unit and the cosines are estimated through

(38) $$\begin{array}{ccc} red\;channel & green\;channel & blue\;channel\\ cos\alpha_{r}=\frac{t_{r1}}{\left|{\overset{⃑}{T}}_{R}\right|} & cos\alpha_{g}=\frac{t_{g1}}{\left|{\overset{⃑}{T}}_{G}\right|} & cos\alpha_{b}=\frac{t_{b1}}{\left|{\overset{⃑}{T}}_{B}\right|}\\ cos\beta_{r}=\frac{t_{r2}}{\left|{\overset{⃑}{T}}_{R}\right|} & cos\beta_{g}=\frac{t_{g2}}{\left|{\overset{⃑}{T}}_{G}\right|} & cos\beta_{b}=\frac{t_{b2}}{\left|{\overset{⃑}{T}}_{B}\right|}\\ cos\gamma_{r}=\frac{\lambda }{\left|{\overset{⃑}{T}}_{R}\right|} & cos\gamma_{g}=\frac{\lambda }{\left|{\overset{⃑}{T}}_{G}\right|} & cos\gamma_{b}=\frac{\lambda }{\left|{\overset{⃑}{T}}_{B}\right|} \end{array}.$$

Satisfying these conditions:

(39) $$\begin{array}{c} {cos}^{2}\alpha_{r}+{cos}^{2}\beta_{r}+{cos}^{2}\gamma_{r}=1\\ {cos}^{2}\alpha_{g}+{cos}^{2}\beta_{g}+{cos}^{2}\gamma_{g}=1\\ {cos}^{2}\alpha_{b}+{cos}^{2}\beta_{b}+{cos}^{2}\gamma_{b}=1 \end{array}.$$

By the analysis, we can determine that noise affects the direction and magnitude of the texture unit, and such changes yield an effect on the classification efficiency of the VIR-TS transform.

#### 2.2.3. Algorithm

This section describes the algorithm to calculate the Vectorial Image Representation on the Texture Space (VIR-TS) transform of a color image and then represent it on the texture space. The algorithm consists of the following steps: (a) the color image S is separated in channels, obtaining the images $S_{R}$, $S_{G}$ and $S_{B}$; (b) three mobile observation windows are defined, $W_{R}=I\times J=3\times 3$, $W_{G}=I\times J=3\times 3$, and $W_{B}=I\times J=3\times 3$; (c) the windows are displaced pixel by pixel across the image under study, with the $W_{R}$ window moving over the $S_{R}$ image, the $W_{G}$ window traversing image $S_{G}$ and the $W_{B}$ windows displacing over the $S_{B}$ image; (d) for each position over the images, the $W_{R}$, $W_{G}$, and $W_{B}$ windows find a pattern $R_{k}=\left[\begin{array}{ccc} r_{11} & r_{12} & r_{13}\\ r_{21} & r_{22} & r_{23}\\ \;r_{31} & r_{32} & r_{33} \end{array}\right]$, $G_{k}=\left[\begin{array}{ccc} g_{11} & g_{12} & g_{13}\\ g_{21} & g_{22} & g_{23}\\ \;g_{31} & g_{32} & g_{33} \end{array}\right]$, $B_{k}=\left[\begin{array}{ccc} b_{11} & b_{12} & b_{13}\\ b_{21} & b_{22} & b_{23}\\ \;b_{31} & b_{32} & b_{33} \end{array}\right]$, where $k=0,1,2,3,\ldots ,N_{p}-1$; (e) the texture units ${\vec{T}}_{R_{k}}$, ${\vec{T}}_{G_{k}}$, ${\vec{T}}_{B_{k}}$ are calculated according to Section 2.2.1 and Section 2.2.2; (f) the procedure is repeated from (a) to (e) for each position on the image under study, all possible texture units ${\overset{⃑}{T}}_{Rk}$, ${\overset{⃑}{T}}_{Gk}$, ${\overset{⃑}{T}}_{Bk}$ are calculated, and thus every color channel is represented by a series of radio vectors [22]; (g) the ${\vec{C}}_{R}$, ${\vec{C}}_{G}$, ${\vec{C}}_{B}$ vectors are calculated, and thus the S image is represented by three vectors where each vector corresponds to an image channel:

(40) $${\overset{⃑}{C}}_{R}=a_{r1}{\hat{u}}_{1}+a_{r2}{\hat{u}}_{2}+{a_{r3}\hat{u}}_{3}$$

(41) $${\overset{⃑}{C}}_{G}=a_{g1}{\hat{u}}_{1}+a_{g2}{\hat{u}}_{2}+{a_{g3}\hat{u}}_{3}.$$

(42) $${\overset{⃑}{C}}_{B}=a_{b1}{\hat{u}}_{1}+a_{b2}{\hat{u}}_{2}+a_{b2}{\hat{u}}_{3}$$

where the components for the vectors, ${\vec{C}}_{R}$, ${\vec{C}}_{G}$, ${\vec{C}}_{B}$, are calculated employing (43).

(43) $$\begin{array}{ccc} red\;channel\;components & green\;channel\;components & blue\;channel\;components\\ a_{r1}=\sum_{k=0}^{N_{P}-1}t_{r1k} & a_{g1}=\sum_{k=0}^{N_{P}-1}t_{g1k} & a_{b1}=\sum_{k=0}^{N_{P}-1}t_{b1k}\\ a_{r2}=\sum_{k=0}^{N_{P}-1}t_{r2k} & a_{g2}=\sum_{k=0}^{N_{P}-1}t_{g2k} & a_{b2}=\sum_{k=0}^{N_{P}-1}t_{b2k}\\ a_{r3}=\sum_{k=0}^{N_{P}-1}t_{r3k} & a_{g3}=\sum_{k=0}^{N_{P}-1}t_{g3k} & a_{b3}=\sum_{k=0}^{N_{P}-1}t_{b3k} \end{array}.$$

**Figure 2:**
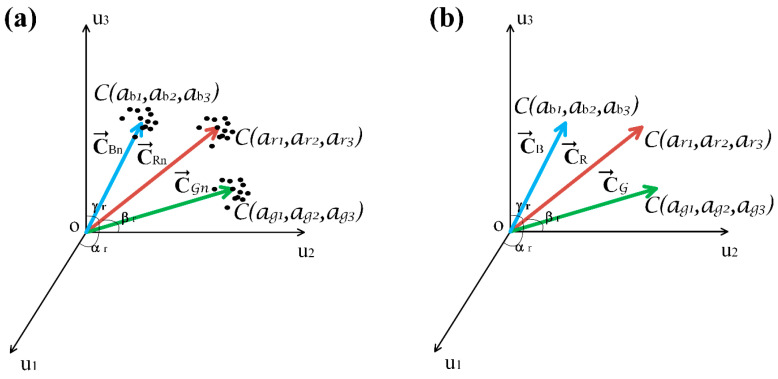
Texture space representation of the RGB image: (**a**) ${\vec{C}}_{Rn}$, ${\vec{C}}_{Gn}$, ${\vec{C}}_{Bn}$ vectors with added noise; (**b**) ${\vec{C}}_{R}$, ${\vec{C}}_{G}$, ${\vec{C}}_{B}$ vectors without present noise.

The total radio vectors will be $N_{P}=\left(M-I+1\right)\times \left(N-J+1\right)$, as was aforementioned. In Figure 2b, the vectors (40)–(42) are represented in the texture space. It can be noted that the vectors yield a unique position because the image is noiseless. In this case, if ${\vec{C}}_{R}$, ${\vec{C}}_{G}$, ${\vec{C}}_{B}$ are deployed as feature vectors in a classifier, the efficiency of the VIR-TS transforms will result in 100%. This maximum efficiency is attributed to the lack of randomness in the bidimensional signal (RGB image).

On the other hand, when an RGB image is acquired through an artificial vision system, this contains noise produced by the light source, photodetection, and the algorithm for signal and instrumentation processing, causing randomness in texture of the units ${\overset{⃑}{T}}_{R}$, ${\overset{⃑}{T}}_{G}$, ${\overset{⃑}{T}}_{B}$; as a consequence, randomness is found in ${\vec{C}}_{R}$, ${\vec{C}}_{G}$, ${\vec{C}}_{B}$. Ergo, when the VIR-TS is calculated for the RGB image, the vectors ${\vec{C}}_{R}$, ${\vec{C}}_{G}$, ${\vec{C}}_{B}$ will wield additional noise, and then they can be expressed through

(44) $${\overset{⃑}{C}}_{Rn}={\overset{⃑}{C}}_{R}+n$$

(45) $${\overset{⃑}{C}}_{Gn}={\overset{⃑}{C}}_{G}+n.$$

(46) $${\overset{⃑}{C}}_{Bn}={\overset{⃑}{C}}_{B}+n$$

where ${\overset{⃑}{C}}_{Rn}$, ${\overset{⃑}{C}}_{Gn},\;{\overset{⃑}{C}}_{Bn}$ are the vectors with noise and n is the random noise of the light source, photodetection, signal and instrumental processing, as was mentioned above. In Figure 2a the three vectors ${\vec{C}}_{Rn}$, ${\vec{C}}_{Gn}$, ${\vec{C}}_{Bn}$ are represented in the texture space, where the black dots are an indicator of a possible random position caused by the noise present in the image. This randomness leads to errors in texture recognition, and consequently, the efficiency of the VIR-TS transform is reduced due to the noise in the RGB image.

### 2.3. Classifier

**Figure 3:**
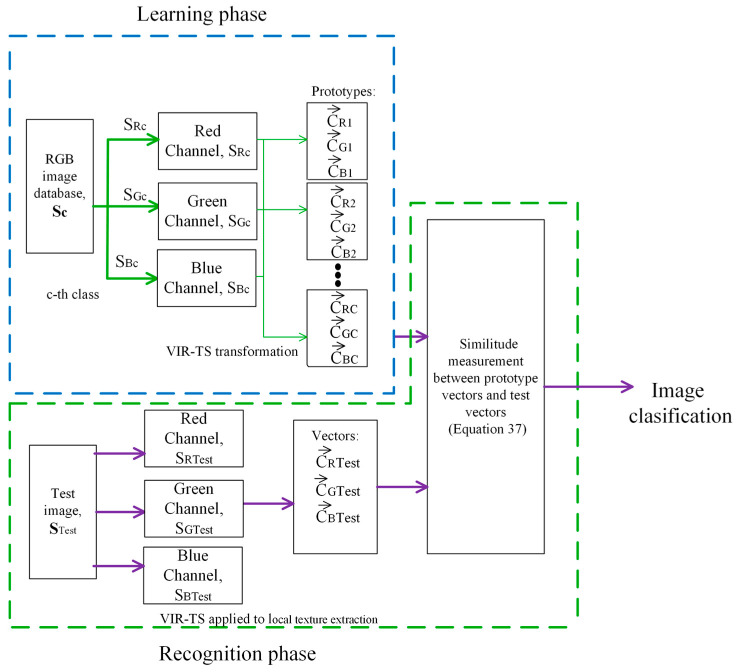
RGB image classifier based on VIR-TS transform.

In reference [22], a multi-class classifier on the texture space was proposed and employed for gray-level image classification. The characteristic vector developed was named $\vec{C}$, which represents a grayscale image in the texture space. This section proposes a scheme deploying this classifier to classify RGB images on the texture space where ${\vec{C}}_{R}$, ${\vec{C}}_{G}$, ${\vec{C}}_{B}$ are employed as feature vectors. Likewise, it consists of two phases: learning and recognition. Figure 3 presents a scheme of its structure.

#### 2.3.1. Learning and Recognition Phases

From Figure 3, in the learning phase prototype vectors are generated from RGB images previously identified by a human expert, $S_{c}\;\left(c=1,2,3,\ldots ,C\right)$, where c−th is the c-nth class and C is the total number of classes. For each class, the ${\vec{C}}_{Rc}$, ${\vec{C}}_{Gc}$, ${\vec{C}}_{Bc}$ vectors are calculated. These vectors are generated with the following procedure: (a) the $S_{c}$ image is separated by its color channels, obtaining separated channel images $\left(S_{Rc},S_{Gc}\;and\;S_{Bc}\right)$ for the c−th class; (b) by applying the algorithm from Section 2.2 to the $S_{Rc}$, $S_{Gc}$ and $S_{Bc}$ images, its VIR-TS transform is calculated and then the prototype vectors for the c−th class are obtained, ${\vec{C}}_{Rc}$, ${\vec{C}}_{Gc}$, ${\vec{C}}_{Bc}$; (c) the procedure indicated in steps a and b is repeated for each class.

In the recognition phase, an unknown test image $S_{Test}$ is separated by its color channels, obtaining $S_{RTest}$, $S_{GTest}$ and $S_{BTest}$ images. (a) Each $S_{RTest}$, $S_{GTest}$ and $S_{BTest}$ image has its VIR-TS transform calculated, and then the ${\vec{C}}_{RTest}$, ${\vec{C}}_{GTest}$, ${\vec{C}}_{BTest}$ vectors are obtained. (b) The similarity between the test image $S_{Test}$ and the prototype image $S_{c}$ is measured in the texture space by means of a projection between vectors:

(47) $$max\left(sim\left(S_{RTest},S_{Rc}\right)\right)=max\left(cos\varphi_{R}\right)=\frac{{\vec{C}}_{RTest}\cdot {\vec{C}}_{Rc},}{\left|{\vec{C}}_{RTest}\right|\left|{\vec{C}}_{Rc}\right|}$$

(48) $$max\left(sim\left(S_{GTest},S_{Gc}\right)\right)=max\left(cos\varphi_{G}\right)=\frac{{\vec{C}}_{GTest}\cdot {\vec{C}}_{Gc},}{\left|{\vec{C}}_{GTest}\right|\left|{\vec{C}}_{Gc}\right|}$$

(49) $$max\left(sim\left(S_{BTest},S_{Bc}\right)\right)=max\left(cos\varphi_{B}\right)=\frac{{\vec{C}}_{BTest}\cdot {\vec{C}}_{Bc},}{\left|{\vec{C}}_{BTest}\right|\left|{\vec{C}}_{Bc}\right|}$$

where $max\left(sim\left(S_{RTest},S_{Rc}\right)\right)$, $max\left(sim\left(S_{GTest},S_{Gc}\right)\right)$, $max\left(sim\left(S_{BTest},S_{Bc}\right)\right)$ is the maximum similarity between images, cos is the trigonometric function cosine, $\varphi_{R}$. $\varphi_{G}$, $\varphi_{B}$ yield the angle formed between vectors, $\left|{\vec{C}}_{RTest}\right|$, $\left|{\vec{C}}_{GTest}\right|$, $\left|{\vec{C}}_{BTest}\right|$ are the vector magnitudes for the test image, $\left|{\vec{C}}_{Rc}\right|$, $\left|{\vec{C}}_{Gc}\right|$, $\left|{\vec{C}}_{Bc}\right|$ represent the magnitude for the prototype images and the dot operator is the scalar product between two vectors. In the classifier, the $S_{Test}$ image is assigned to a c class if and only if the projection between the test vectors and the prototype is close to the unit (or it is the unit).

#### 2.3.2. Decision Criteria

Given that the RGB images are classified by color channels, the classifier yields three possible Boolean outputs: $A_{R}=1$ or $A_{R}=0$ is the acceptance or rejection of the red channel, $A_{G}=1$ or $A_{G}=0$ is the acceptance or rejection of the green channel and $A_{B}=1$ or $A_{B}=0$ is the acceptance or rejection of the blue channel. If the classifier outputs are considered Boolean variables, their possible combinations are in concordance with Table 1, where all input variables are $A_{R}$, $A_{G}$, $A_{B}$, and the output variable, $O_{D}$, is the classifier’s decision.

Based on Table 1, two cases are considered.

Case I: The classifier has a rigorous criterion if and only if the condition $A_{R}=1$,$\;A_{G}=1$,${\;A}_{B}=1$ is satisfied.

Case II: The classifier has a not rigorous criterion if and only if the images of two channels are accepted and then the decision of the classifier satisfies the Boolean equation

(50) $$O_{D}=\bar{A_{R}}A_{G}A_{B}+A_{R}\bar{A_{G}}A_{B}+A_{R}A_{G}\bar{A_{B}}$$

Applying Boolean algebra, Equation (32) can be reduced to

(51) $$O_{D}=A_{G}A_{B}+A_{R}A_{B}+A_{R}A_{G}$$

Equation (33) becomes the decision criterion of the multi-class classifier when two color channels are classified correctly and one is rejected.

#### 2.3.3. Texture Descriptors and Classifiers

##### LBPs (Local Binary Patterns)

**Figure 4:**
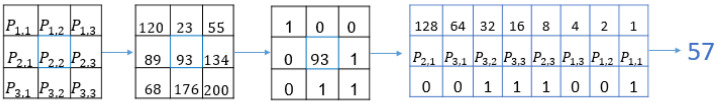
LBP descriptor.

LBPs are designed to describe grayscale images employing an observation window of 3 × 3 to obtain numeric values between 0 and 255 considering the patterns $P_{i,j}=[P_{1,1},P_{2,1},P_{3,1},P_{3,2},P_{3,3},P_{2,3},P_{1,3},P_{1,2},P_{2,2}]$, where $P_{2,2}$ is the central pixel (see Figure 4); this descriptor compares $P_{2,2}$ against each of its neighboring pixels $P=[P_{2,1},P_{3,1},P_{3,2},P_{3,3},P_{2,3},P_{1,3},P_{1,2},P_{1,1}]$. If the condition $P_{i,j}$ ≥ $P_{2,2}$ is satisfied, a 1 is assigned, otherwise, $P_{i,j}$ < $P_{2,2}$, and a 0 is assigned instead. An 8-bit binary value is generated, which is later converted to its respective decimal value between 0 and 255, which is stored in the resulting feature vector of $R^{256}$ [23].

##### CS_LBPs (Center-Symmetric Local Binary Patterns)

**Figure 5:**
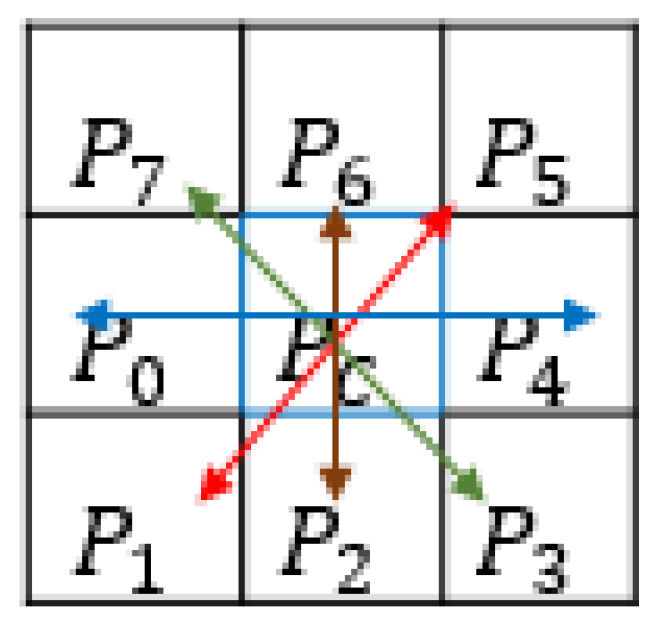
Symmetric pairs.

CS_LBPs are descriptors that, contrary to LBPs, discard the central pixel $P_{C}$; instead, pairs of symmetric pixel values are considered by ignoring the center: ($P_{0},P_{4}$), ($P_{1},P_{5}$), ($\;P_{3},P_{7}$), ($\;P_{2},P_{6}$) (see Figure 5).

A threshold is applied to the gray tone differences employing the parameter Δ; then, a set of $2^{4}$ patterns is generated. Its kernel function is expressed by

(52) $$f(x)=\sum_{j=0}^{3}2^{j}s(P_{j}-P_{j+4}-\Delta -1)$$

where $P_{j}$ represents the intensity of the neighbor, $P_{j+4}$ represents the intensity of the symmetric neighbor and s corresponds to the threshold function defined by

(53) $$s\left(x\right)=\left\{\begin{array}{c} 1,\;x\geq \Delta \\ 0,\;x<\Delta \end{array}\right.$$

As a result, 4 bits are extracted, defining a dimensional space of $R^{16}$ [24].

##### SVM (Support Vector Machine) Classifier

SVM is a statistical classifier commonly deployed for image classification. Give a class c composed by a set of images of a single individual, a texture descriptor (such as LBP or CS_LBP) is applied to generate a vector with textural characteristics, which is represented by x, where $x_{i=}\left\{x_{1},x_{2},x_{3},\ldots ,x_{i}\right\}$; the class labels are defined by $y_{l}\in \left\{-1,\;1\right\}$, becoming $y_{l=}\left\{y_{1},y_{2},y_{3},\ldots ,y_{l}\right\}$. Equation (36) defines the hyperplane:

(54) w×x+b=0

Considering (w,b) of the image $x_{i}$, it can be described with Equation (37):

(55) fxi=signw×x+b=1    yl=1−1    yl=−1

where $y_{l}\;$ determines the class c of the image x [25].

##### Random Forest Classifier

Random Forest is a popular classifier used to perform the regression and classification of datasets. It builds multiple individual classifiers to generate a prediction. Given a set of data $x_{i=}\left\{x_{1},x_{2},x_{3},\ldots ,x_{i}\right\}$, where $y_{l=}\left\{y_{1},y_{2},y_{3},\ldots ,y_{l}\right\}$ represents the classes c [26], the classifier determines to which class c the set of data x belongs.

## 3. Experimental Work

Numerical experiments are presented in this section. The image classification efficiency is tested for the VIR-TS transform, and the database employed contains face images, which were previously classified by a human expert. For the first experiment, an image is selected from the database, and random noise is added to it; hence, when the VIR-TS transform is calculated, ${\vec{C}}_{R}$, ${\vec{C}}_{G}$, ${\vec{C}}_{B}$ and ${\vec{C}}_{Rn}$, ${\vec{C}}_{Gn}$, ${\vec{C}}_{Bn}$ vectors have different positions due to noise. For the second experiment, the image database is recognized according to the classifier described in Section 2.3. In this experiment random noise is not added; therefore, the efficiency of the VIR-TS transform without additional noise can be verified. For the third experiment, the classification efficiency of the VIR-TS transform is studied considering the effects of noise. The results are presented in a behavior graphic of efficiency vs. SNR. For experiments 2 and 3, the decision criterion is rigorous, called case 1.

### 3.1. Database

**Figure 6:**
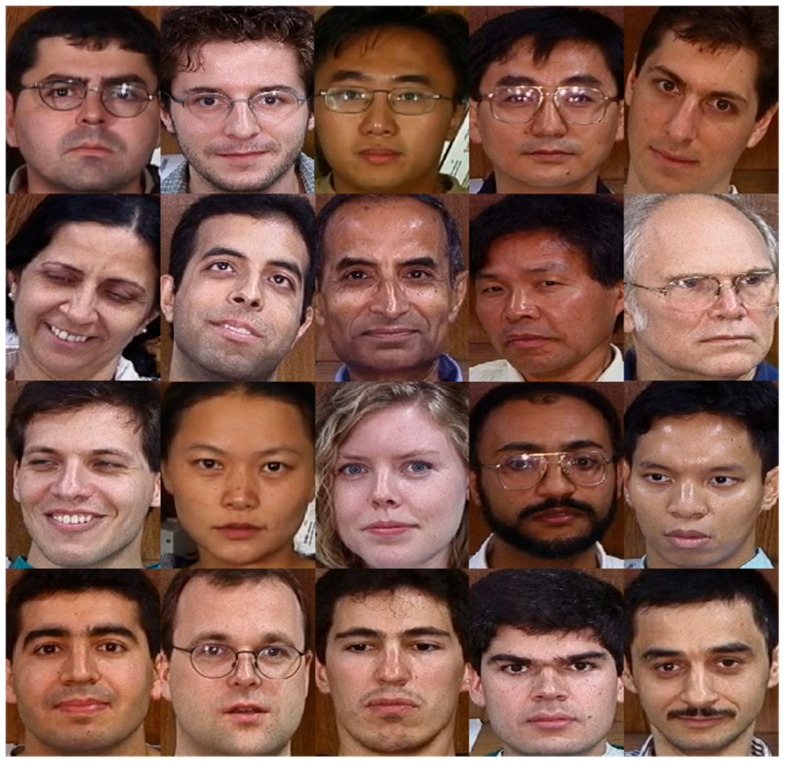
Collage of selected images [27].

The Georgia Tech (GT) database was employed [27]. It contains images captured by the signal and image processing center of the Georgia Institute of Technology. Each individual had 15 images taken in uncontrolled environments; thus, some have frontal or tilted faces, with different facial expressions, in addition to different lighting and scale and with an approximate resolution of 150 × 150 pixels. To perform the tests of this paper, 20 pictures (shown in Figure 6) were selected, where each individual image is considered as a class.

### 3.2. FEI Database

The FEI Database consists of a collection of photographs of 50 different individuals, where each individual has a set of 15 pictures. This database was compiled by the Artificial Intelligence Laboratory of FEI in São Bernardo do Campo, São Paulo, Brazil [28].

### 3.3. Experiment 1: Effect of Noise on ${\vec{C}}_{R}$, ${\vec{C}}_{G}$, ${\vec{C}}_{B}$ Vectors

**Figure 7:**
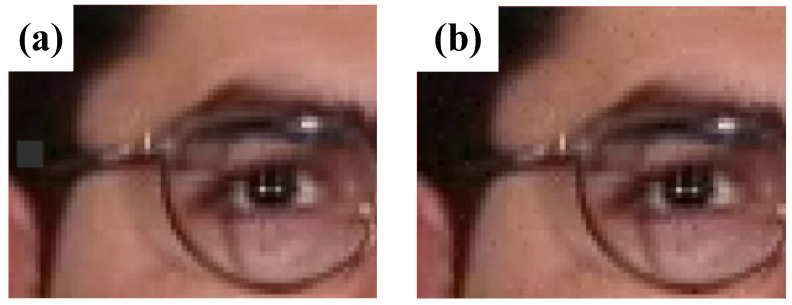
Segment of class 3: (**a**) RGB image without additional noise; (**b**) RGB image with noise: σ=6 and (signal-to-noise ratio) SNR=33.85.

Based on reference [29], Gaussian distributed noise was added to the color image. The procedure is described below: (a) image S in RGB format is taken as input; (b) the RGB image is divided in the channels that compose it, generating the $S_{R}$, $S_{G}$ and $S_{B}$ images, where $S_{R}$ is the red channel image, $S_{G}$ is the green channel image and $S_{B}$ is the blue channel image; (c) random noise with Gaussian distribution, σ = 6, is generated; (d) the noise is added to 10% of each $S_{R}$, $S_{G}$ and $S_{B}$ image, where n is the noise in the color image, $n_{r}$ is the noise added to the red channel, $n_{g}$ is the noise added to the green channel and, lastly, $n_{b}$ is the noise added to the blue channel, resulting in $S_{R}+n_{r}$, $S_{G}+n_{g}$ and $S_{B}+n_{b}$. Figure 7a presents the RGB image with no additional noise, and Figure 7b presents the result after the addition of noise: σ=6 and (signal-to-noise ratio) SNR=33.85.

**Figure 8:**
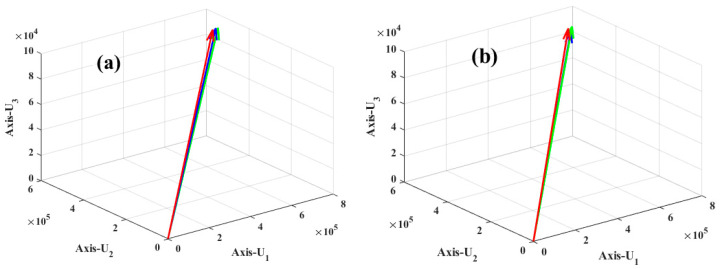
Texture space representation of class 3: (**a**) ${\vec{C}}_{R}$, ${\vec{C}}_{G}$, ${\vec{C}}_{B}$ vectors; (**b**) ${\vec{C}}_{Rn}$, ${\vec{C}}_{Gn}$, ${\vec{C}}_{Bn}$ vectors.

Following the procedure described in Section 2.2, the VIR-TS transform was calculated for class 3, obtaining the result in Figure 8. Figure 8a presents the ${\vec{C}}_{R}$, ${\vec{C}}_{G}$, ${\vec{C}}_{B}$ vectors when class 3 has no additional noise. Meanwhile, Figure 8b presents the ${\vec{C}}_{Rn}$, ${\vec{C}}_{Gn}$, ${\vec{C}}_{Bn}$ vectors, but in this case, class 3 has noise. Comparing Figure 8a,b, when noise is added to the RGB image in every channel, the vector position changes in the texture space, increasing its randomness. This can be observed in Table 2, where the values of the ${\vec{C}}_{R}$, ${\vec{C}}_{G}$, ${\vec{C}}_{B}$, ${\vec{C}}_{Rn}$, ${\vec{C}}_{Gn}$, ${\vec{C}}_{Bn}$ vectors are displayed: Column 1 shows class 3; Column 2 presents the component values of the ${\vec{C}}_{R}$ and ${\vec{C}}_{Rn}$ vectors; Column 3 displays the component values of the ${\vec{C}}_{G}$ and ${\vec{C}}_{Gn}$ vectors; and last, Column 4 shows the component values for the ${\vec{C}}_{B}$ and ${\vec{C}}_{Bn}$ vectors.

Analyzing Figure 8 and Table 2, on the texture space, the ${\vec{C}}_{R}$, ${\vec{C}}_{G}$, ${\vec{C}}_{B}$ vectors have a different position from the ${\vec{C}}_{Rn}$, ${\vec{C}}_{Gn}$, ${\vec{C}}_{Bn}$ vectors due to the noise added to class 3, causing a greater randomness in the ${\vec{C}}_{Rn}$, ${\vec{C}}_{Gn}$, ${\vec{C}}_{Bn}$ vectors. These position changes account for errors in image classification when the VIR-TS transform is employed for recognition.

### 3.4. Experiment 2: Image Classification of RGB Images Without Added Noise

This section verifies the efficiency of the VIR-TS transform when random noise is not added to any of the 20 classes, as shown in Figure 6. The multi-class classifier was described in Section 2.3, and its characteristic vector is defined by three (${\vec{C}}_{R}$, ${\vec{C}}_{G}$, ${\vec{C}}_{B}$) vectors. In the multi-class classifier, each RGB image is considered a class, and the ${\vec{C}}_{R}$, ${\vec{C}}_{G}$, ${\vec{C}}_{B}$ vectors are calculated for each $S_{c}$ class with the following procedure: (a) the c−th class is separated by channels, obtaining the $S_{Rc}$, $S_{Gc}$ and $S_{Bc}$ images; (b) the VIR-TS transform is calculated for the $S_{Rc}$, $S_{Gc}$ and $S_{Bc}$ images, resulting in the ${\vec{C}}_{Rc}$, ${\vec{C}}_{Gc}$, ${\vec{C}}_{Bc}$ vectors; (c) steps a and b are repeated for each class until all prototypes are generated for the classifier; (d) a test image $S_{Test}$ is divided in channels to generate the $S_{RTest}$, $S_{GTest}$ and $S_{BTest}$ images; (e) each $S_{RTest}$, $S_{GTest}$ and $S_{BTest}$ image has its VIR-TS transform calculated to obtain the ${\vec{C}}_{RTest}$, ${\vec{C}}_{GTest}$, ${\vec{C}}_{BTest}$ vectors; (f) the resulting ${\vec{C}}_{RTest}$, ${\vec{C}}_{GTest}$, ${\vec{C}}_{BTest}$ vectors are compared against the prototype vectors, ${\vec{C}}_{Rc}$, ${\vec{C}}_{Gc}$, ${\vec{C}}_{Bc}$, where the similarity between vectors is calculated by means of vector projection; (g) the test image $S_{Test}$ is assigned to a c−th class based on Equation (8), i.e., the vectors that bear the most similarity on the texture space.

#### Evaluation Metrics

The effectiveness of the classification model is measured to evaluate the efficiency, taking into account the following [30,31]: TP, correctly classified positives; TN, correctly classified negatives; FP, negatives classified as positives; and FN, positives classified as negatives.

Precision identifies positive simples of the positive prediction results.

(56) $$Precision=\frac{TP}{TP+FP}$$

Accuracy identifies the correctly classified classes through all the predictions.

(57) $$Accuracy=\frac{TN+TP}{TP+TN+FP+FN}$$

Recall identifies correct predictions of the positive results.

(58) $$Recall=\frac{TP}{TP+FN}$$

F1-Score identifies the mean harmonic between recall and precision.

(59) $$F1-score=2\times \frac{Precision\times Recall}{Precision+Recall}$$

**Figure 9:**
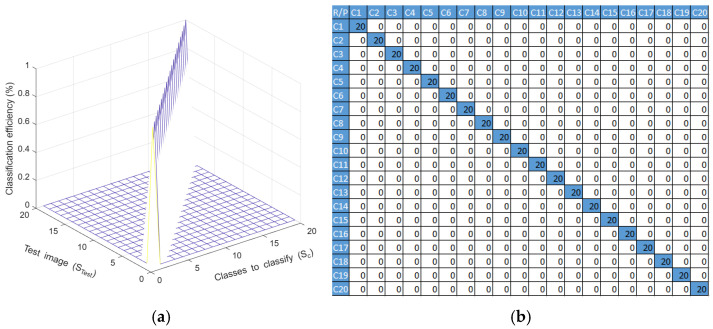
(**a**) Image classification results implementing VIR-TS transforms when classes have no added noise. (**b**) Confusion matrix.

The image classification results were obtained through a confusion matrix where the successes appear in the main diagonal and the classification errors are the elements outside of the scope of the main diagonal. Figure 9 illustrates the classification efficiency of the VIR-TS transform if the classes have no added noise.

In Figure 9, the classification efficiency can be calculated with

(60) $${Ef}_{\%}=\frac{1}{C}\sum_{c}diag(M)\times 100$$

where ${Ef}_{\%}$ is the classification efficiency in a percentage, M is the confusion matrix, $\sum_{c}diag(M)$ is the sum of all elements within the confusion matrix and C is the total of classes from the image database.

(61) $${Ef}_{\%}=\frac{1+1+1+1+1+1+1+1+1+1+1+1+1+1+1+1+1+1+1+1}{20}\times 100$$

The efficiency, EF, is 100% when noise is not added to the classes. Hence, we can confirm that the VIR-TS transform is efficient in image recognition applications; nonetheless, its effectiveness is due to the sensibility of random noises in the classes.

Table 3 displays a comparative study to verify the evaluation metrics of the descriptors and classifiers proposed in this paper against LBP and CS_LBP descriptors with the SVM and RF classifiers. It can be noted that the proposed technique surpasses the results in all the measured metrics.

### 3.5. Experiment 3: Classification of RGB Images with Added Noise

**Figure 10:**
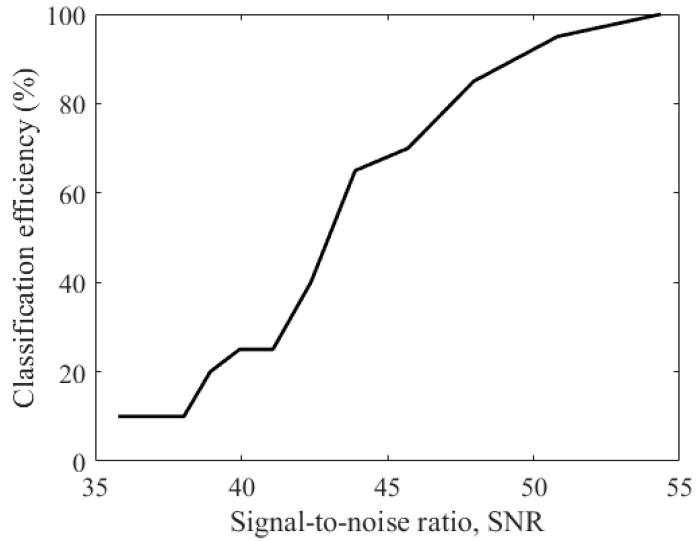
Behavior graphic: classification efficiency vs. signal-to-noise ratio.

In this section, the face images presented in Figure 6 are classified with the VIR-TS transform after each class received a Gaussian distributed random noise addition in 10% of their pixels. The images were catalogued by employing the multi-class classifier described in Section 2.3. The procedure is detailed as follows: (a) each class $S_{c}$ was separated by its color channels, $S_{Rc}$, $S_{Gc}$ and $S_{Bc}$, and each extracted image had a Gaussian distributed random noise added to them, obtaining $S_{Rc}+n_{r},\;S_{Gc}{+n}_{g},\;S_{Bc}{+n}_{b}$ as a result; (b) from each image 100 sub-images with randomly added Gaussian noise were extracted, obtaining a total of 5000 images from the 50 original ones; (c) afterwards, a mean was calculated for each image and the VIR-TS transform was applied to it, obtaining the ${\vec{C}}_{Rc}$, ${\vec{C}}_{Gc}$, ${\vec{C}}_{Bc}$ vectors for the c−th class; (d) an $S_{Test}$ test image was acquired and separated by the component channels to generate $S_{RTest}$, $S_{GTest}$ and $S_{BTest}$; (e) the VIR-TS transform was calculated for the $S_{RTest}$, $S_{GTest}$ and $S_{BTest}$ images, and, consequently, the ${\vec{C}}_{RTest}$, ${\vec{C}}_{GTest}$, ${\vec{C}}_{BTest}$ vectors were obtained; (f) the ${\vec{C}}_{Rc}$, ${\vec{C}}_{Gc}$, ${\vec{C}}_{Bc}$ prototype vectors and the ${\vec{C}}_{RTest}$, ${\vec{C}}_{GTest}$, ${\vec{C}}_{BTest}$ vectors were compared; then, the $S_{Test}$ test image was assigned to the class it was closer to. The similarity between classes was measured by means of vector projection on the texture space, as indicated in Equation (31). Figure 10 illustrates the behavior of classification efficiency (%) vs. the signal-to-noise ratio.

In Figure 10, for SNR=54.36 or greater, the VIR-TS transform presents an efficiency of 100%, and this value is equal to the results obtained with images with no added noise, as can be seen in the results in experiment 2. Under these conditions, the classes do not have enough noise to disturb the position of the ${\vec{C}}_{Rc}$, ${\vec{C}}_{Gc}$, ${\vec{C}}_{Bc}$ vectors on the texture space. Contrary to this, if the SNR value diminishes due to an increment of the noise in the class, then the classification decreases given the randomness of the ${\vec{C}}_{Rc}$, ${\vec{C}}_{Gc}$, ${\vec{C}}_{Bc}$ vectors higher on the texture space. Ergo, the vision systems built through the VIR-TS transform yield an efficiency lower than 100%.

#### Training and Testing Classification Procedure with Noise (Figure 11)

The model of the sub-images without added noise is generated as follows:

1. Take an image of each class $S_{c}$.
2. Generate sub-images.
3. Apply a texture descriptor (proposed, LBP or CS_LBP) to generate the feature vectors.
4. Train the model with 80% of the generated sub-images.
5. Perform a classification procedure with the remaining 20% with the proposed classifier, SVM and RF.
6. Generate the trained model.

Evaluation of the classification procedure of sub-images with noise was performed as follows:

7. Take an image from each class $S_{c}$ and add a Gaussian noise with the function np.random.normal of the NumPy library and a parameter of σ = 6, resulting in SNR = 38.3947.
8. Generate sub-images from the images with added noise.
9. Implement the descriptors (proposed, LBP or CS_LBP) to generate the feature vectors.
10. Train the model employing the feature vectors generated with noise as input.
11. Predict which class the studied sub-image belongs to.

**Figure 11:**
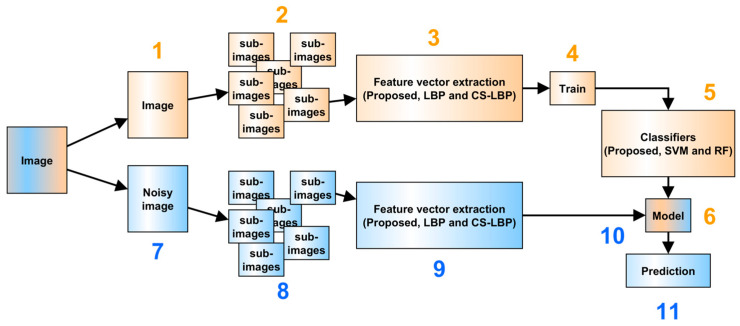
Training and testing procedure.

The procedure described in Training and Testing Classification Procedure with Noise (Figure 11) was performed to evaluate the classification accuracy. Table 4 presents a comparative study where the evaluation metrics with descriptors proposed in this paper are measured considering the LBP and CS_LBP descriptors and the SVM and Random Forest classifiers. From the experimental results reported in Table 4, VIR-TS is better that LBP-SVM and CS-LBP, but its efficiency is lower than the LBP-RF. Therefore, our proposal can be applied because its efficiency is higher that some texture extraction techniques reported.

## 4. Discussion

In this paper, facial RGB images are classified on the texture space of the Vectorial Image Representation on the Texture Space (VIR-TS) transform. The RGB images were identified based on the color compositions of their channels given that each channel was represented by a vector, ${\vec{C}}_{R}$, ${\vec{C}}_{G}$, ${\vec{C}}_{B}$. Here, ${\vec{C}}_{R}$, ${\vec{C}}_{G}$, ${\vec{C}}_{B}$ were employed as characteristic vectors in a multi-class classifier, and the similarity between classes was measured by means of a projection between prototype vectors and test vectors. Experimentally, the classification efficiency of the VIR-TS transform was evaluated. Based on theoretical analysis and experimental results, the following points are worth noting:

(a) The VIR-TS transform can classify RGB images on texture space, performing a recognition based on the color channels.
(b) The VIR-TS transform returns a 100% efficiency in image identification when the classes have no added noise and if the signal-to-noise ratio is a greater value.
(c) By reducing the signal-to noise ratio in the class, the efficiency of the VIR-TS transform decreases due to the increment of the randomness in the position of the ${\vec{C}}_{Rn}$, ${\vec{C}}_{Gn}$, ${\vec{C}}_{Bn}$ vectors on the texture space.
(d) Efficient artificial vision systems can be built based on the VIR-TS transform if and only if the SNR is high.
(e) The VIR-TS transform is not invariant to scale or rotation, and these effects on the class could reduce its image classification efficiency.
(f) The VIR-TS transform can be applied in security systems, medical diagnosis, image reconstruction, and locating people, among other applications.
(g) The VIR-TS is founded by concepts of linear algebra, as it is a homogeneous system of equations based on a pattern P detected by the observation window I×J=3×3 (mm). On the other hand, other texture extraction techniques (LBP and CCR) are founded in probability and statistic concepts; ergo, the resulting texture unit is employed as an index for discrete histograms.
(h) The foundation of the VIR-TS transform is based in linear algebra, while other transforms are founded in filtering processes (texture extraction techniques by signal processing), statistical events (statistical texture extraction techniques), fractals and/or polynomials (texture extraction techniques using mathematical models). Thus, the VIR-TS technique is a novel methodology used to represent the texture data, and this representation is, in fact, a texture space.

Comparing the VIR-TS technique against the statistical texture extraction techniques (LBP and CCR), the VIR-TS represents an RGB image through three vectors, ${\vec{C}}_{R}$, ${\vec{C}}_{G}$, and ${\vec{C}}_{B}$, where each vector corresponds to a color channel with three components. Meanwhile, the statistical techniques generate a discrete histogram, $p\left(k\right)\;\left(\;k=0.1.2,\ldots ,K-1\right)$, with a K length depending on the codification method and the observation window size I×J. Considering the aforementioned, our method offers advantages over the statistical techniques because the computer operations required for classification are significantly reduced when two images are compared. This reduction in computer operations is noticeable when the similarity between two images is measured: VIR-TS measures the similarity comparing three vectors, ${\vec{C}}_{R}$, ${\vec{C}}_{G}$, ${\vec{C}}_{B}$, while the LBP and CCR techniques measure the similarity with discrete histograms [23], rendering the possibility of a buffer overflow.

Comparing our image classification proposal with the one reported in reference [22], both papers performed image classification employing the VIR-TS transform. However, our proposal applies the VIR-TS transform in RGB images where classes received random noise to demonstrate its high classification efficiency. Furthermore, comparing the VIR-TS transform with the techniques reported in references [22,32,33], our proposal analyzes the texture through a transformation, but in the references the texture is studied by its frequency components. Conversely, comparing our proposal with references [34,35,36,37], our proposal is founded in linear algebra, and the literature analyzes the texture with statistical fundaments. Considering the aforementioned, our future work has the following lines of interest: (a) study the invariance to rotation and scale for the VIR-TS transform; (b) implement artificial vision systems based on the VIR-TS transform; (c) develop a mathematic formality for the VIR-TS transform; (d) develop statistical analysis regarding the efficiency and behavior of classifiers versus noise; (e) improve the classification efficiency of the VIR-TS with techniques based on noise reduction.

## 5. Conclusions

In this paper, facial recognition was implemented by employing RGB images. Each color channel had its local texture characteristics extracted with the Vectorial Image Representation on the Texture Space (VIR-TS) transform, representing every RGB image by three random vectors, ${\vec{C}}_{R}$, ${\vec{C}}_{G}$, and ${\vec{C}}_{B}$, on the texture space. Experimentally, by deploying a multi-class classifier, the efficiency of the VIR-TS transform was corroborated considering two cases: without noise added to the class and with noise added to the class. In the first case, when an image had no added noise, the VIR-TS transform returned an efficiency of 100%. For the second case, when the face images had noise, the efficiency returned 100% if and only if the SNR=50.36 or greater; otherwise, the efficiency decreased proportionally to the lower value of the SNR.

Based on these results, the VIR-TS transform can be implemented to design artificial vision systems with high efficiency. It can be applied in medical diagnosis, security, access control, medical image analysis, image reconstruction, and locating people, to mention some examples.

Our future work will consist of strengthening and improving this area of research to further our contribution with techniques to allow the descriptor to work with images in uncontrolled environments that present different degrees of rotation, brightness and occlusion.

## Figures and Tables

**Table 1:** Decision criteria employed in the texture space multi-class classifier.

Classifier Output by Channel			Classification Decision	Case
$A_{R}$	$A_{R}$	$A_{R}$	$A_{R}$	Not applicable
0	0	0	0	Not applicable
0	0	1	0	Not applicable
0	1	0	0	Not applicable
0	1	1	1	Not rigorous (2)
1	0	0	0	Not applicable
1	0	1	1	Not rigorous (2)
1	1	0	1	Not rigorous (2)
1	1	1	1	Rigorous (1)

Note: 1 indicates acceptance and 0 indicates rejection.

**Table 2:** Vector positions in the texture space when class 3 has no additional noise and with additional noise.

Class 3	${\vec{C}}_{R}$, ${\vec{C}}_{G}$, ${\vec{C}}_{B}$ vectors calculated for class 3 without additional noise								
	${\vec{C}}_{R}$			${\vec{C}}_{G}$			${\vec{C}}_{B}$		
	Components			Components			Components		
	$t_{r1}$	$t_{r2}$	$t_{r3}$	$t_{g1}$	$t_{g2}$	$t_{g3}$	$t_{b1}$	$t_{b2}$	$t_{b3}$
	802,100	549,400	105,600	852,300	570,400	103,300	833,500	563,800	104,400
	${\vec{C}}_{Rn}$, ${\vec{C}}_{Gn}$, ${\vec{C}}_{Bn}$ vectors calculated for class 3 with additional noise								
	${\vec{C}}_{Rn}$			${\vec{C}}_{Gn}$			${\vec{C}}_{Bn}$		
	Components			Components			Components		
	$t_{r1}+n_{r}$	$t_{r2}+n_{r}$	$t_{r3}+n_{r}$	$t_{g1}+n_{g}$	$t_{g2}+n_{g}$	$t_{g3}+n_{g}$	$t_{b1}+n_{b}$	$t_{b2}+n_{b}$	$t_{b3}+n_{b}$
	724,200	528,100	109,700	764,900	548,900	107,900	754,300	545,400	104,400

**Table 3:** Classification comparison between the different descriptors and classifiers.

		FEI Face DB	GT Face DB
Proposed	Accuracy	100	100
	Precision	100	100
	Recall	100	100
	F1-Score	100	100
LBP-SVM	Accuracy	80.00	80.00
	Precision	70.00	70.00
	Recall	80.00	80.00
	F1-Score	73.33	73.33
CS-LBP-SVM	Accuracy	80.00	80.00
	Precision	70.00	70.00
	Recall	80.00	80.00
	F1-Score	73.33	73.33
LBP-RF	Accuracy	80.00	80.00
	Precision	71.67	70.00
	Recall	80.00	80.00
	F1-Score	74.17	73.33
CS-LBP-RF	Accuracy	80.00	80.00
	Precision	71.67	70.00
	Recall	80.00	80.00
	F1-Score	74.17	73.33

**Table 4:** Comparison of evaluation metrics.

		FEI Face DB	GT Face DB
		Noise	Noise
Proposed	Accuracy	60.00	80.00
	Precision	47.17	72.00
	Recall	60.00	80.00
	F1-Score	51.13	74.67
LBP-SVM	Accuracy	5.64	91.98
	Precision	1.11	92.29
	Recall	5.64	91.98
	F1-Score	1.49	91.94
CS-LBP-SVM	Accuracy	14.54	76.24
	Precision	19.02	77.99
	Recall	14.54	76.24
	F1-Score	10.12	75.87
LBP-RF	Accuracy	17.38	98.64
	Precision	4.33	98.66
	Recall	17.38	98.64
	F1-Score	6.58	98.64
CS-LBP-RF	Accuracy	96.80	98.88
	Precision	96.84	98.89
	Recall	96.80	98.88
	F1-Score	96.80	98.88

## Data Availability

The data related to the results that support our conclusions are available upon request to the authors.
